# Exposure to household air pollutants and endothelial dysfunction in rural Bangladesh: A cross-sectional study

**DOI:** 10.1097/EE9.0000000000000132

**Published:** 2021-02-19

**Authors:** Mohammad Hasan Shahriar, Muhammad Ashique Haider Chowdhury, Shyfuddin Ahmed, Mahbubul Eunus, Shirmin Bintay Kader, Bilkis A. Begum, Tariqul Islam, Golam Sarwar, Rabab Al Shams, Rubhana Raqib, Dewan S. Alam, Faruque Parvez, Habibul Ahsan, Md Yunus

**Affiliations:** a Department of Public Health Sciences, Biological Science Division, The University of Chicago, Chicago, Illinois; b UChicago Research Bangladesh, Dhaka, Bangladesh; c icddr,b, Dhaka, Bangladesh; d Robert Stempel College of Public Health and Social Work, Florida International University, Miami, Florida; e Chemistry Division, Atomic Energy Center, Dhaka, Bangladesh; f School of Kinesiology and Health Sciences, Faculty of Health, York University, Toronto, Ontario, Canada; g Mailman School of Public Health, Columbia University, New York, New York; h Institute for Population and Precision Health, The University of Chicago, Chicago, Illinois

**Keywords:** Household air pollution, Endothelial dysfunction, Particulate matter, Reactive hyperemia index, icddrb, URB

## Abstract

More than one third of world’s population use biomass fuel for cooking that has been linked to an array of adverse health hazards including cardiovascular mortality and morbidity. As part of Bangladesh Global Environmental and Occupational Health (GEO Health) project, we assessed whether household air pollution (HAP) was associated with dysfunction in microvascular circulation (measured by reactive hyperemia index [RHI]).

**Methods:**

We measured exposure to HAP (particulate matter [PM2.5], carbon monoxide [CO], and black carbon [BC]) for 48 hours of 200 healthy nonsmoker adult females who used biomass fuel for cooking. Exposure to PM2.5 and BC were measured using personal monitor, RTI MicroPEM (RTI International, NC) with an internal filter that had been both pre- and post-weighed to capture the deposited pollutants concentration. Lascar CO logger was used to measure CO. Endothelial function was measured by forearm blood flow dilatation response to brachial artery occlusion using RHI based on peripheral artery tonometry. A low RHI score (<1.67) indicates impaired endothelial function.

**Results:**

Average 48 hours personal exposure to PM2.5 and BC were 144.15 μg/m^3^ (SD 61.26) and 6.35 μg/m^3^ (SD 2.18), respectively. Interquartile range for CO was 0.73 ppm (0.62–1.35 ppm). Mean logarithm of RHI (LnRHI) was 0.57 in current data. No statistically significant association was observed for LnRHI with PM2.5 (odds ratio [OR] = 0.97; 95% confidence interval [CI] = 0.92, 1.01; *P* = 0.16), BC (OR = 0.85; 95% CI = 0.72, 1.01; *P* = 0.07), and CO (OR = 0.89; 95% CI = 0.64, 1.25; *P* = 0.53) after adjusting for potential covariates.

**Conclusions:**

In conclusion, HAP was not associated with endothelial dysfunction among nonsmoking females in rural Bangladesh who used biomass fuel for cooking for years.

**What this study adds**

Our exposure assessment method was robust since we measured exposure for 48 hours twice a year. Also, our outcome measure was novel in context of environmental epidemiology, especially while measuring as an effect of household air pollution (HAP) (this is the first study to evaluate the HAP effect on endothelial dysfunction so far our knowledge). Although we did not observe any association, this finding is significant because we measure exposure at personal level which indicates that the association we observed was relatively unbiased.

## Introduction

Air pollution is a major public health problem for last few decades. Concentrations of household air pollution (HAP) from biomass smoke (wood, dung, agricultural residue, etc.) have been associated with an array of health outcomes including cardiopulmonary morbidity and all-cause mortality.^1–4^ About one third of world population use biomass fuel for cooking^4^ and a growing number of US population (about 2 million) use it for heating their homes.^5^ The recent World Health Organization (WHO) report on the Global Burden of Disease estimates that over 3.5 million annual deaths and 110 million disability-adjusted life years (DALYs) were linked to biomass use in 2010.^4,6^ Most recent reports showed almost similar trends in death and DALYs, but the death occurs at much younger age.^4^ This mortality burden from HAP has doubled over the last decade and a half. The mortality outcomes are predominantly due to cardiovascular disease and pulmonary effects endpoints in adults.^4^

Evidence is accumulating in support of the role of outdoor air pollution and cardiovascular disease-related outcomes^7–10^ such as endothelial dysfunction.^11,12^ Nitric oxide (NO) plays many roles in maintaining vascular health, most importantly its role in vasomotor tone. When the bioavailability of NO is reduced for some reason, it leads to impairment of endothelium-dependent vasodilation which is called endothelial dysfunction. The ultimate consequence of endothelial dysfunction is the formation of atherosclerosis and its late sequelae, cardiovascular morbidity, and mortality followed by an inflammatory process.^13–20^ That’s the reason researchers defined it as “ultimate risk of the risk factors” for cardiovascular disease.^15^

Ambient and traffic-related air pollutions have been found to be associated with endothelial dysfunction.^12,21–23^ A number of population studies have found outdoor air pollutants including particulate matter (PM) and black carbon (BC) to be associated with vascular endothelial function assessed by flow-mediated dilation (FMD).^22,24–27^ In the Multi-Ethnic Study of Atherosclerosis (MESA) cohort, Krishnan et al^28^ found an annual increase of PM2.5 by 3 μg/m^3^ to be associated with a 0.3% reduction in FMD. Interestingly, two other studies have observed an improved endothelial function with a reduction in reactive hyperemia-peripheral arterial tonometry (RH-PAT) among healthy individuals following use of air filtration.^29,30^ However, very little data exist from population-based studies on the current risk estimates for biomass induced cardiovascular disease (CVD) outcomes. The limited evidence comes from small studies that have considered non-HAP sources of air pollution. For instance, a recent report found a higher prevalence of carotid plaques (odds ratio [OR] = 2.6; *P* = 0.03) and increased carotid intima-media thickness (IMT), a marker of atherosclerosis that is predictive of adverse health events^31^ among biomass users compared with clean fuel users (mean difference = 0.03 mm; *P* = 0.02).^32,33^ Among a small sample of improved stoves users (N = 49) in Guatemala, a reduction of abnormal electro cardiogram was observed (OR = 0.26; 95% confidence interval [CI] = 0.08, 0.90 for ST-segment depression) as compared to users of traditional stove (N = 70).^34^ A recent study found that particles (PM2.5) levels were correlated with an increase in cardiovascular events in healthy women.^35^ This study suggested that air pollution affects patients with preexisting cardiovascular disease and healthy subjects. Taken together, these studies indicate a link between outdoor air pollution and impaired endothelial function and other preclinical measures of CVD; however, such effects have not been evaluated in HAP (biomass) exposed population with similar type of pollutants (PM2.5, BC) as outdoor air pollution. Also, most of earlier studies measured exposure from monitoring station and/or from kitchen monitor rather than at personal level.^12,20–22,32,33^ The proposed study was the first to investigate effects of individual-level measurement of exposure to multiple components of HAP on an established preclinical marker of CVD peripheral artery endothelial dysfunction.

Noninvasive measurements of endothelial function have been used extensively in vascular research,^36^ which is also easier to conduct and more compliant than invasive method. Flow-mediated dilation (FMD) assessed by brachial artery ultrasound is the most frequently applied method^37–40^, but special training is required since it is highly dependent on operator experience level to obtain accurate measurement.^41^ Other widely used noninvasive method is pulse amplitude tonometry (PAT) in the index finger after reactive hyperemia, which is easy accessible method^42–45^ and need little training to operate. Also, it is well validated both in healthy individuals^46–49^ and in patients with coronary artery disease (CAD).^50^ In this current study, we used RH-PAT considering the above-mentioned facility (less operator experience-dependent and training) over FMD.

## Methods

### Study design

We conducted a cross-sectional study to assess the associations of HAP with a preclinical maker of CVD, reactive hyperemia index (RHI). HAP exposure had been assessed for PM2.5, carbon monoxide (CO), and BC by collecting personal air samples for 48-hour samples from 200 participants.

### Study site

This study was implemented in Matlab and Araihazar subdistricts under Chandpur and Narayanganj districts, respectively, in Bangladesh. Matlab is located about 57 km and Araihazar is 34 km from Dhaka (the capital city of Bangladesh). The International Center for Diarrheal Disease and Research, Bangladesh (icddr,b) maintains Matlab field site covering a current population of ~225,826 in 142 villages and UChicago Research Bangladesh maintains a parent cohort study (Health Effects of Arsenic Longitudinal Study [HEALS]), which included ~35,000 married adults at Araihazar field site.^51,52^

### Study population (eligibility and exclusion criteria)

**Figure 1.**
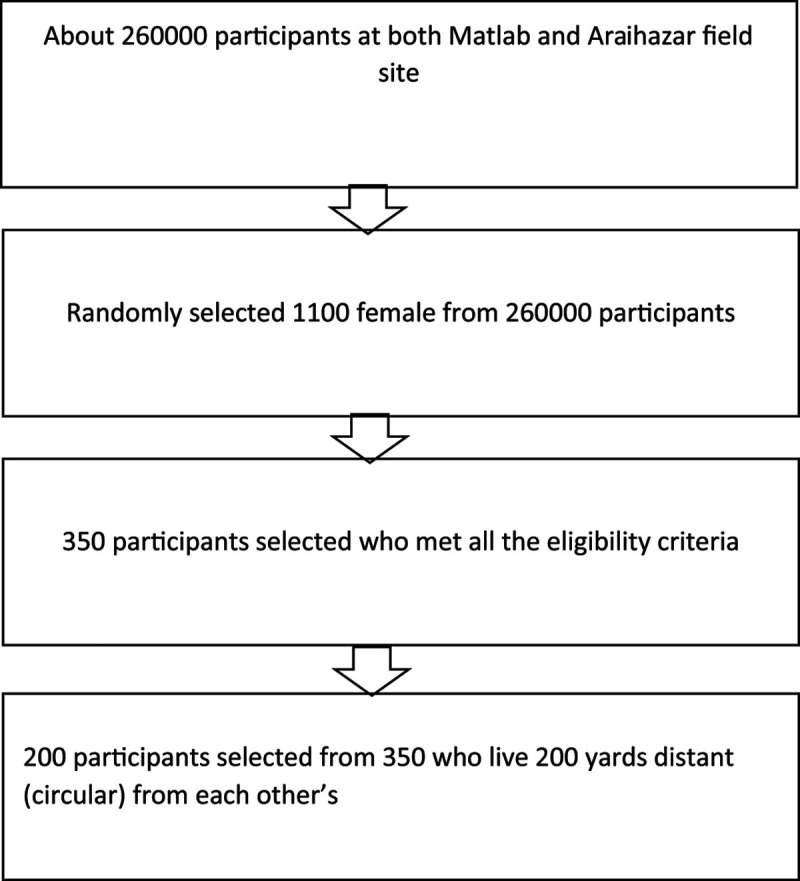
Participants selection flow chart.

We randomly selected 1,100 female participants who used biomass fuel for cooking and then 350 participants from 1,100 who met the following eligibility criteria: (1) between 25 and 65 years old, (2) live in homes with biomass burning traditional stoves, (3) nonsmoker and live with nonsmokers, (4) exposed to <10 μg/L of water arsenic, and (5) not known to have any clinical events of CVD or lung disease, including stroke or coronary heart disease. Finally, we selected 200 adult females living within 200 yards of circular distance from a selected participant’s household to avoid contamination of exposure between households (Fig. 1). Only one woman was included per Bari (i.e., a cluster of households for the extended family). Since smoking and arsenic in drinking water may affect some of the study outcomes, we restricted among nonsmoking individuals who drink water containing <10 μg/L of arsenic to avoid their potential confounding effects.

### Recruitment procedure

A field team in each site was involved in collecting air samples and interview data. Our central data center generated a list of potential participants for the study using the selection criteria as described. The study team contacted each eligible participant, described the study procedure, and ascertained eligibility criteria. If the participant was willing, then the study team made a schedule for a convenient time for air monitoring. The study supervisor was responsible for preparing the equipment before deployment, downloading data after deployment, changing filters, and routine maintenance of the air monitors. The village health workers deployed the personal air samplers on the subjects and collected daily questionnaire data (see below). An appointment was arranged for the subject at the health center for outcome assessment.

### Exposure assessment—questionnaire data

We collected information on other potential determinants including fuel usage pattern, number and types of stoves used, types of food cooked, kitchen type (enclosed, semienclosed, open), distance to living quarter, ventilation in both kitchen and home, home lighting sources, and other sources of exposures, etc. This data was used for improving exposure models. Questionnaire was validated and used in other earlier studies in Bangladesh.^53,54^

### Exposure assessment—HAP

Each participant in the study was asked to carry two small exposure monitors (CO and PM2.5/BC, see below) for 48 hours twice a year at least 6 months apart for taking care of the seasonal variation in exposure. The monitors were placed in small, culturally appropriate bags that were placed near the breathing zone. The total weight of the two monitors was less than 2 lbs. We had daily dairy to enlist activities within 48 hours of air sample collection. If anybody of them outside their home premises, we could track their outdoor activities. We found 99% of the participants are housewife, and they resided home almost 24/7 during our sampling period. They did not visit relatives or elsewhere far from their home. Smith et al^55^ suggested a single 72 hours monitoring period is sufficient for estimating the true long-term mean exposure.^56^ However, we were able to measure for 48 hours as our devices stopped between 48 hours and 60 hours of running due to power exhaustion. We were unable to detect the exact reason but manufacturer and other experts assumed that the filter blocked with carbon particles at high pollution levels and the air pump exhausted the battery power (three lithium-ion AA batteries) trying to maintain the flow rate (~0.4 L/min). Therefore, our measurement might not reflect the long-term measurement in one sense in terms of duration of measurement as referenced above, but as we measured 48 hours for twice in two different seasons that might reflect the long-term measurement.

#### PM2.5 and BC

PM2.5 and BC were measured using an RTI MicroPEM (RTI International, NC) (version3.2). The range of detection of the monitor is 1 to 10,000 mg/m^3^.^57^ The MicroPEM (RTI International) measured the contaminants continuously using light scattering principles and also gravimetrically with an internal filter that was pre- and post-weighed in a temperature- and humidity-controlled room on a microbalance with accuracy of 1 μg in the Atomic Energy Centre, Dhaka (AECD) laboratory. We weighted the filters before and after exposure using a microbalance (METTLER Model MT5) by maintaining room temperature at 22 °C and relative humidity at 50% to determine the fine mass of PM. Before every weighing, we equilibrated the samples of fine fractions at constant humidity and temperature of the balance room. To eliminate the static charge accumulated on the filters before each weighing, we used STATICMASTER, a U-shape electrostatic charge eliminator. We used appropriate laboratory and field blanks to ensure the quality control of filter weight.

To measure BC, we analyzed the PM2.5 samples by a well calibrated EEL-type Smoke Stain Reflectometer.^58^ The amount of reflected light that was absorbed by the filter sample and an assumed mass absorption coefficient defined the concentrations. It is related to the concentration of light-absorbing carbon through standards of carbon with known areal density. BC value measured by reflectance negligibly influenced by iron (Fe), which has a moderate light absorption coefficient. The influence of variation in Fe concentration on BC measurement was neglected as the uncertainty associated with the BC measurement was rather high (4%–9%). We used 10 m^2^/g, the extinction coefficient, for the BC calculation.^58^

#### Carbon monoxide

CO was monitored using the Lascar EL-USB-CO data logger. These low-cost sensors are small, lightweight, and able to measure at a 1-ppm resolution between 0 and 1,000 ppm continuously at a 1-minute time resolution.

#### Quality assessment/quality control

All real-time PM2.5 data were corrected for zero drift based on high efficiency particulate air filtered air measurements at the beginning and end of each deployment and then normalized to the ratio of gravimetric filter to the mean of the real-time data during active time period of sampling. Wearing compliance of MicroPEM (RTI International) device was assessed by analyzing the accelerometer (a quality control tool inside the MicroPEM, RTI International) data logged by the MicroPEM (RTI International). The accelerometer was sensitive enough to sense breathing movement even if the participant was sitting still.

### Reactive hyperemia-peripheral arterial tonometry

RH-PAT is considered a marker of endothelial function. RH-PAT has been associated with CVD risk^59^ and CVD risk factors in the Framingham study.^37,60^ The measure has been validated as reliable in healthy adults.^48^ RH-PAT measured by EndoPAT has been validated with coronary microvascular function (potentially induced by particulate matter associated thromboinflammatory responses)^61,62^ and has been reported to have a high reproducibility (intraclass correlation = 0.75).^48^

For the current study, RH-PAT was measured with the finger plethysmographic device (EndoPAT2000; Itamar Medical Ltd, Caesarea, Israel) placed on the tip of each index finger, as previously described by Bonetti et al.^63^ In short, we used the PAT device on the tip of each index finger to measure pulse volume changes and expressed them as pulse amplitude. We measured it in the fingertips of both hands for 2 minutes and 20 seconds at baseline. Then we calculated the PAT ratio (the ratio of the post deflation pulse amplitude [90–120 seconds after deflation] to the baseline preocclusion pulse amplitude of the same finger) by inflated forearm cuff at 250 mmHg on one arm for 5 minutes to induce ischemia. We followed similar method for the control finger of the opposite arm to get a PAT ratio and divided by the PAT ratio of test finger to produce the “reactive hyperemia index (RHI).” For analyses, we used natural logarithm of RHI (LnRHI) since the distribution was skewed. A lower RHI value (<1.67 for RHI or <0.51 for LnRHI) indicates impaired hyperemic response to ischemia.^64^

### Ethical approval

Our study involves human subjects. The research protocol was approved (protocol number: PR-15111) by icrdrb Research Review Committee (RRC) and Ethical Review Committee (ERC) and all human participants gave written informed consent. All relevant necessary documents have been attached with the article.

### Statistical analysis

We examined the distributions of the variables and calculated descriptive statistics, for the overall sample and by group of biomass exposure. In bivariate analyses, we used chi-square tests to detect group differences for categorical variables; *t* test for two-group comparisons for continuous variables with symmetric distributions and approximately equal within-group variances and Wilcoxon rank test for asymmetric distribution; scatter plots and Spearman correlation to examine bivariate relationships between continuous variables, especially between air pollutants and LnRHI. To assess the association between air pollutant exposures and specific RHI measures, we used logistic regression models (LnRHI <0.51 as endothelial dysfunction, which was coded as 1 and LnRHI ≥0.51 as normal, which was coded as 0). We used LnRHI (based on the distribution) to assess the associations with each of the air pollutants (i.e., PM2.5, BC, and CO), adjusting for the potential confounding variables (age, body mass index [BMI], education, household income, cooking duration [years of cooking with biomass fuel], systolic blood pressure [SBP], and diastolic blood pressure [DBP]). The confounding variables were selected based on known predictors of outcome (i.e., measures for endothelial dysfunction) and their potential relationship with HAP. Since all participants were female, unexposed to tobacco and arsenic, we avoided their confounding effects. Finally, since we measured exposure at different times of the year, we explored the substantial seasonal variability in air pollutants between dry (November to April) and wet (May to October) seasons. Differential susceptibility of menopause and obesity were investigated by stratifying age (≤51 years and >51 years) and BMI (<25 and ≥25). We also tested for interaction of HAP measures with age, BMI, SBP, and DBP as continuous variables. Log likelihood ratio (LR) test was used to ascertain the model fit.

We used nonparametric kernel regression as well to assess the effect of HAP (PM2.5 and CO) on endothelial function as parametric regression model did not fit the data (*F* statistics > 0.05) even after transforming the exposure and outcome variables (variables were not normally distributed). We used simple linear regression for BC after log transformation. We preferred to report results from the logistic regression model here since other models did not show different results than logistic model; also, a well-established cutoff for LnRHI is evident^64^ and easier interpretation of results for readers.

In addition, we performed sensitivity analysis collapsing the LnRHI < 0.30 as endothelial dysfunction and LnRHI ≥ 0.30 as normal endothelial function since that cutoff had 80% sensitivity and 85% specificity as evidenced from Bonetti et al.^61^

## Results

Table 1 shows the characteristics of the study participants. Mean RHI level for the participants was 1.77. Less than 0.51 for LnRHI (RHI 1.67) indicates impaired hyperemic response to ischemia^64^ and 42.71% of participants had their LnRHI values less than 0.51.

**Table 1.** Characteristics of study participants

Characteristics (n = 199)	Mean ± SD	LnRHI **≤** 0.51 (n = 84)	LnRHI > 0.51 (n = 115)	*P*
Age (years)	38 ± 7.5	38.25 ± 7.24	37.61 ± 7.74	0.69
Age (years)				
≤44	74%			
>44	26%			
BMI	24.26 ± 4.27	23.83 ± 4.52	24.58 ± 4.07	0.22
BMI				
<25	60.50%			
≥25	39.50%			
Education (years)	5 ± 3	5.15 ± 3.40	5.00 ± 3.58	0.74
Education (years)				
No education	19%			
At least primary level and above	81%			
Household income (BDT)	10,193 ± 7,268	10,324.71 ± 7,610.22	10,097.35 ± 7,100.55	0.00
Years exposed to biomass fuel (IQR)	16 (11.5–22)	17 (12–20)	16 (11–25)	0.61
Daily cooking time (IQR) (hours)	2 (2–3)	2 (2–3)	3 (2–3)	0.00
Heart rate (beats/min)	77.4 ± 11.46	78.89 ± 11.07	76.29 ± 11.72	0.11
SBP (mmHg)	112.78 ± 12.76	112.85 ± 11.57	112.73 ± 13.62	0.94
DBP (mmHg)	74.77 ± 9.47	74.11 ± 8.12	75.26 ± 10.38	0.40
Exposure to air pollutants (48 hours)				
PM2.5 (μgm^–3^)	144.14 ± 61.26	138.59 ± 58.45	143.46 ± 50.24	0.53
Black carbon (μgm^–3^)	6.35 ± 2.18	6.74 ± 2.22	5.82 ± 2.03	0.00
CO (ppm) (IQR)	0.97 (0.62–1.35)	0.90 (0.65–1.23)	0.99 (0.62–1.45)	0.16

A lower RHI value (<1.67 for RHI or <0.51 for LnRHI) indicates impaired hyperemic response to ischemia or endothelial dysfunction (Source: ITAMAR Website).

BDT indicates Bangladeshi Taka/month; IQR, interquartile range.

Mean PM2.5 concentration based on gravimetric methods were 144.15 μgm^–3^, which was several folds higher than the WHO limit (<25 μgm^–3^ for 24 hours).^65^ Mean BC and CO concentrations were 6.35 μgm^–3^ and 1.15 ppm, respectively, which were lower than the WHO recommended values (<25 μgm^–3^ and 7 ppm).^65^ Statistically significant mean difference of HAP was observed between season 1 (dry) and season 2 (wet) except for CO (Table 2).

**Table 2.** Seasonal variation of HAP

HAPs	Season 1	Season 2	*P*
	Mean ± SD (mg/m^3^)	Mean ± SD (mg/m^3^)	
PM2.5	126.42	161.88	0.00
BC	5.34	7.37	0.00
CO	1.12	1.18	0.77

Season 1 was dry season (November to April), and season 2 was wet season (May to October).

**Figure 2.**
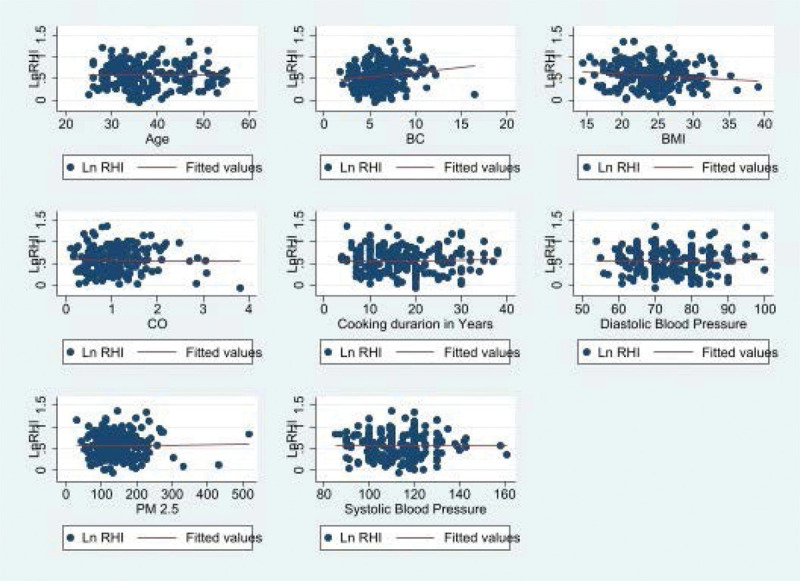
Scatter plot showing association between RHI and age, BMI, cooking duration (hours/day), DBP, SBP, and years of HAP use.

Scatter plot of age and cooking duration (years), systolic blood pressure, diastolic blood pressure, PM2.5, and CO showed no correlation with LnRHI; however, BMI showed a negative and BC showed a positive correlation with LnRHI (Fig. 2). In addition, the correlation matrix showed that none of the pollutants were strongly correlated among themselves except for systolic and diastolic blood pressure that were strongly correlated. All of the pollutants were negatively correlated with LnRHI, but the correlation was very weak (Table 3).

**Table 3.** Spearman correlation of LnRHI with air pollutants and covariates

	SBP	DBP	PM2.5 (LS)	CO	PM2.5 (GM)	BC	LnRHI
SBP	1.0000						
DBP	0.7098	1.0000					
PM2.5 (LS)	0.1486	0.0721	1.0000				
CO	0.0629	0.1311	0.0379	1.0000			
PM2.5 (GM)	–0.0721	0.0357	0.0606	0.2144	1.0000		
BC	–0.0436	0.1421	0.1422	0.2648	0.3983	1.0000	
LnRHI	0.0299	–0.0283	–0.0214	–0.0707	–0.0439	–0.2096	1.0000

GM indicates gravimetric; LS, light scattering.

The mean LnRHI for the participants was 0.57 (SD = 0.28). No significant association was observed between LnRHI and HAP–PM2.5 (OR = 0.97; 95% CI = 0.92, 1.01; *P* = 0.16), BC (OR = 0.85; 95% CI = 0.72, 1.01; *P* = 0.07), and CO (OR = 0.89; 95% CI = 0.64, 1.25; *P* = 0.53) after adjusting for covariates (Table 4).

**Table 4.** Effect of PM2.5, black carbon, and CO exposure on LnRHI

Exposure	OR (95% CI)	*P*
PM2.5	0.97 (0.92, 1.01)	0.16
Black carbon	0.85 (0.72, 1.01)	0.07
Carbon monoxide	0.89 (0.64, 1.25)	0.53

Models were adjusted for age, BMI, education, household income, cooking duration, SBP, and DBP. Separate pollutant models were run.

We found no evidence that the associations between LnRHI and HAP varied by age and BMI, which indicate that menopause and obesity did not have any effect on the observed associations (Table 5). Effect estimates for HAP measures on endothelial function were almost identical across seasons, and there were no significant variations (Table 6). We also observed the PM2.5 effect to be modified by SBP and the model with the interaction terms had a better fit based on LR test. DBP was not significantly associated with LnRHI (OR = 1.10; CI = 0.95, 1.27; *P* = 0.17). However, SBP was significantly associated with LnRHI but the association was protective (OR = 0.89; CI = 0.84, 0.99; *P* = 0.04) and the interaction term with PM2.5 had no effect (OR = 1.00; CI = 1.00, 1.00; *P* = 0.02) on LnRHI (results not shown).

**Table 5.** Differential effect of PM2.5, black carbon, and CO exposure on LnRHI by menopause and obesity

Exposure	OR (95% CI)	*P*
PM2.5	0.97 (0.93, 1.02)	0.29
Black carbon	0.50 (0.14, 1.82)	0.30
Carbon monoxide	0.85 (0.51, 1.45)	0.57

Separate pollutant models were run.

**Table 6.** Effect estimates of HAP on endothelial function by seasonal variation

Air pollutants	Season 1		Season 2	
	OR (95% CI)	*P*	OR (95% CI)	*P*
PM2.5	1.00 (0.96, 1.04)	0.84	1.00 (0.99, 1.01)	0.26
BC	3.24 (0.82, 12.86)	0.10	0.90 (0.82, 1.00)	0.07
CO	0.98 (0.67, 1.43)	0.92	0.90 (0.67, 1.20)	0.47

Season 1 was dry season (November to April), and season 2 was wet season (May to October).

In nonparametric kernel regression models (since multiple linear regression model did not fit our data even after transformation of exposure and outcome variables in case of PM and CO models), we did not find any associations between HAP and endothelial dysfunction (Table 7). In case of BC, we found protective association with endothelial dysfunction in simple linear regression model; however, the R-squared value was very low, 0.03 (not shown). In sensitivity analysis (LnRHI < 0.30 and LnRHI ≥ 0.30), we also did not find any associations between HAP and endothelial dysfunction (Table 8).

**Table 7.** Effect of PM2.5 and CO exposure on LnRHI (nonparametric Kernel regression)

Exposure	β (95% CI)	*P*
PM2.5	0.0005084 (–0.0006848, 0.0012203)	0.29
Carbon monoxide	0.0281313 (–0.0343798, 1.333526)	0.55

Separate pollutant models were run.

**Table 8.** Effect of PM2.5, black carbon, and CO exposure on LnRHI (**≤**0.30 and >0.30)

Exposure	OR (95% CI)	*P*
PM2.5	1.27 (0.53, 3.05)	0.59
Black carbon	0.57 (0.20, 1.64)	0.30
Carbon monoxide	1.10 (0.62, 2.00)	0.73

Models were adjusted for age, BMI, education, household income, cooking duration, SBP, and DBP. Separate pollutant models were run.

## Discussion

In this cross-sectional study, we observed average exposure to PM2.5 among the participants to be much higher than WHO recommended levels in rural nonsmoking women in Bangladesh. However, we did not find any association between LnRHI, a measure of endothelial function with any of the HAP measures PM2.5, BC, and CO after adjustment for possible confounding variables. Seasonal variability in HAP did not have any influence on these associations. In addition, since all of the participants were women, normotensive, nondiabetic, nonsmoker, and exposed to low level of arsenic through drinking water (<10 ppb), it is unlikely that known extraneous factors had any impact on the observed associations.

It is well established that hypertension is associated with endothelial dysfunction^66–68^, although it is still unclear whether endothelial dysfunction is a cause or an effect of hypertension. We also found association between hypertension and endothelial dysfunction but only for SBP (OR = 0.89; CI = 0.84, 0.99; *P* = 0.04) while we included it in the PM model. DBP was not significantly associated with endothelial dysfunction (OR = 1.10; CI = 0.95, 1.27; *P* = 0.17). However, Bedirian et al^69^ found diastolic function were correlated with endothelial dysfunction among diabetic and hypertensive group of people. Further studies are needed to explore this differential effects.

The evidence of the effects of air pollution on endothelial function is equivocal. A small study (42 healthy white) in Paris in 2007 found no association between air pollutants and endothelium-independent glycerin trinitrate (GTN)-induced brachial artery dilatation.^24^ Exposure to particle rich or particle filtered air did not influence microvascular function (MVF) or the inflammatory biomarkers significantly as evidenced from a randomized crossover study. They let the recruited people exercise for 180 minutes (with or without biking) and with 24-hour exposure to particle rich air. Relatively low concentration of PM (24 μg/m^3^) and younger aged people (blood vessels of young aged group may not as sensitive as to cause deleterious effects from air pollution exposure) could be a possible explanation for this insignificant response in MVF and biomarkers.^30^ O’Neill et al^26^ found accordingly that impairment of vascular reactivity at ambient levels only susceptible to a specific group of people (diabetic and urban people).

Framingham Heart Study Offspring and Third Generation Cohorts investigated the effect of ambient air pollution on microvessel function measured by peripheral arterial tonometry in 2008. The mean LnRHI were 0.57 ± 0.38 for men and 0.83 ± 0.40 for women. They did not find any association between averaging periods of PM2.5, black carbon, particle number, sulfate, and nitrogen oxides and endothelial dysfunction. Unexpected higher LnRHI with higher exposure was observed for longer moving averages of air pollutants. No changes of the results were noted even after exclusion of the 3 days when air pollution levels were higher than normal for 24-hour PM2.5_,_ current smokers, and systolic blood pressure from the model in sensitivity analysis. The outcome of this study was the effect of short-term exposure to air pollutants that support our current study findings.^70^

In contrast, in 2012, a large cohort study called Multi-Ethnic Study of Atherosclerosis and ambient Air Pollution (MESA Air) aimed to investigate whether long- and short-term exposure to PM2.5 had an effect on decreased flow-mediated dilatation (FMD) and/or decreased brachial artery distensibility (BAD). They found an annual increase of PM2.5 by 3 μg/m^3^ to be associated with a 0.3% reduction in FMD (95% CI of difference = –0.6, –0.03; *P* = 0.03) and short-term variation in PM2.5 was not significantly associated with endothelial dysfunction.^23^ These two study findings indicated that short-term exposure to air pollutants was not associated with endothelial dysfunction, which explained our null finding as well.

A group of researchers from Harvard and Boston University back in 2011 showed that medium-term exposure (12 weeks average) was positively associated with markers of inflammation and endothelial dysfunction, and this effect was more pronounced among diabetic and nonstatin user group.^22^ Two other studies found endothelial dysfunction due to diluted diesel exhaust exposure and arterial vasoconstriction without endothelial dysfunction due to concentrated ambient fine particles plus ozone exposure.^71,72^

Animal experiments also found mixed findings. While systemic administration of diesel exhaust particles, a decreased vasodilation (acetylcholine-induced) in aortic rings was observed among hyperlipidemic apoE knockout mice and increased response was observed among wild-type mice.^73^

Major strengths of the study include rigorous control of confounding factors (through restriction and statistical models), well-characterized study population, and rigorous multimodal exposure assessments. There are many reasons that may explain inconsistent results among different studies including different methods to assess microvascular function, air pollutants composition, co-pollutants, host susceptibility, different study population, exposure error, and duration and frequency of exposure assessment. For our study, the absence of association between HAP and endothelial dysfunction may be due to the cross-section design, modest sample size, relatively younger age of participants, and absence of HAP effect on small vessels unlike brachial arteries.^74,75^ We did not adjust for physical activity, lipid profile, and nutritional factors in our analyses, although such factors were unlikely to confound our observed study associations. Finally, while RH-PAT is a noninvasive method based on small vessels with little operator dependence, this is not the gold standard for measuring endothelial dysfunction. Future studies need to evaluate other methods and tools for measuring endothelial dysfunction in relation to HAP.

In conclusion, this cross-sectional study did not find any association of HAP constituents PM2.5, BC, and CO with peripheral small vessel endothelial function (measured by EndoPAT) in rural nonsmoking females in Bangladesh. Future studies need to investigate these associations using larger sample size and prospective design and also potentially other methods of endothelial function measurement.

## Conflicts of interest statement

The authors declare that they have no conflicts of interest with regard to the content of this report.

The results reported herein correspond to specific aims of grant U01TW010120 and U2R TW010122 to investigators (H.A. and M.Y.) from Fogarty International Center of the National Institutes of Health (NIH). This work was also supported by grant R24 ES028532 to investigator (H.A.) from NIH. Data and code can be obtained from corresponding author via email.

The content is solely the responsibility of the authors and does not necessarily represent the official views of the National Institutes of Health.

## ACKNOWLEDGMENTS

International Center for Diarrheal Disease and Research, Bangladesh (icddr,b) acknowledges with gratitude the commitment of National Institutes of Health to its research efforts. icddr,b is also grateful to the Governments of Bangladesh, Canada, Sweden, and the United Kingdom for providing core/unrestricted support. We would like to thank all of our participants, research staff, Ryan Cartier, and specialist from RTI International.
